# Storage Time and DNA Quality Determine *BRCA1/2* Sequencing Success in Prostate Cancer: A Multicentre Analysis with Therapeutic Implications

**DOI:** 10.3390/cancers17101705

**Published:** 2025-05-20

**Authors:** Mariavittoria Vescovo, Maria Rosaria Raspollini, Lorenzo Nibid, Francesca Castiglione, Eleonora Nardi, Dario de Biase, Francesco Massari, Francesca Giunchi, Francesco Pepe, Giancarlo Troncone, Umberto Malapelle, Mariantonia Carosi, Beatrice Casini, Elisa Melucci, Matteo Fassan, Luisa Toffolatti, Elena Guerini-Rocco, Federica Conversano, Alessandra Rappa, Stefania Tommasi, Claudio Antonio Coppola, Pio Zeppa, Alessandro Caputo, Sara Gaeta, Fabio Pagni, Davide Seminati, Andrea Vecchione, Stefania Scarpino, Daniela Righi, Chiara Taffon, Francesco Prata, Giuseppe Perrone

**Affiliations:** 1 Anatomical Pathology Operative Research Unit, Fondazione Policlinico Universitario Campus Bio-Medico, Via Alvaro del Portillo, 200, 00128 Rome, Italy; lorenzo.nibid@unicampus.it (L.N.); d.righi@policlinicocampus.it (D.R.); c.taffon@policlinicocampus.it (C.T.); g.perrone@policlinicocampus.it (G.P.); 2 Histopathology and Molecular Diagnostics, University Hospital Careggi, Via Pieraccini, 6, 50129 Florence, Italy; mariarosaria.raspollini@aouc.unifi.it (M.R.R.); francesca.castiglione@gmail.com (F.C.); eleonora.nardi@unifi.it (E.N.); 3 Research Unit of Anatomical Pathology, Department of Medicine and Surgery, Università Campus Bio-Medico di Roma, Via Alvaro del Portillo, 21, 00128 Rome, Italy; 4 Solid Tumor Molecular Pathology Laboratory, IRCCS Azienda Ospedaliero-Universitaria di Bologna, 40138 Bologna, Italy; dario.debiase@unibo.it; 5 Department of Pharmacy and Biotechnology, University of Bologna, 40126 Bologna, Italy; 6 Medical Oncology, IRCCS Azienda Ospedaliero-Universitaria di Bologna, 40138 Bologna, Italy; francesco.massari8@unibo.it; 7 Department of Medical and Surgical Sciences (DIMEC), University of Bologna, 40138 Bologna, Italy; 8 Pathology Unit, IRCCS Azienda Ospedaliero-Universitaria di Bologna, 40138 Bologna, Italy; francesca.giunchi@aosp.bo.it; 9 Department of Public Health, University Federico II of Naples, 80131 Naples, Italy; pepefrancesco88@yahoo.it (F.P.); giancarlo.troncone@unina.it (G.T.); umbertomalapelle@gmail.com (U.M.); 10 Molecular Diagnostic Laboratory, Pathology Department, Advanced Diagnostics Research and Technological Innovation Department, IRCCS Regina Elena National Cancer Institute, Via Elio Chianesi, 53, 00144 Rome, Italy; mariantonia.carosi@ifo.it (M.C.); beatrice.casini@ifo.it (B.C.); elisa.melucci@ifo.it (E.M.); 11 Department of Medicine (DIMED), University of Padua, 35122 Padua, Italy; matteo.fassan@gmail.com; 12 Veneto Institute of Oncology (IOV-IRCCS), 35128 Padua, Italy; 13 Surgical Pathology Unit, Ca’ Foncello General Hospital, ULSS2 Marca Trevigiana, 31100 Treviso, Italy; luisa.toffolatti@aulss2.veneto.it; 14 Department of Oncology and Hemato-Oncology, University of Milan, 20122 Milan, Italy; elena.guerinirocco@ieo.it; 15 Division of Pathology, European Institute of Oncology, IRCCS, 20141 Milan, Italy; federica.conversano@ieo.it (F.C.); alessandra.rappa@ieo.it (A.R.); 16 Pharmacogenetics and Molecular Diagnostics Unit, IRCCS Istituto Tumori Giovanni Paolo II Bari, 70124 Bari, Italy; stefania.tommasi@gmail.com (S.T.); c.a.coppola@oncologico.bari.it (C.A.C.); 17 Department of Medicine, Surgery, and Dentistry “Scuola Medica Salernitana”, University of Salerno, 84084 Baronissi, Italy; p.zeppa@unisa.it (P.Z.); alessandro.caputo94@gmail.com (A.C.); 18 Department of Pathology, University Hospital of Salerno, 84081 Salerno, Italy; sara.gaeta@sangiovannieruggi.it; 19 Department of Medicine and Surgery, University Milan Bicocca and Fondazione IRCCS San Gerardo dei Tintori Monza, 20126 Milan, Italy; fabio.pagni@unimib.it (F.P.); 20 Unit of Pathology, Department of Clinical and Molecular Medicine, Sant’Andrea Hospital, Sapienza University, 00189 Rome, Italy; andrea.vecchione@uniroma1.it (A.V.); stefania.scarpino@uniroma1.it (S.S.); 21 Department of Urology, Fondazione Policlinico Universitario Campus Bio-Medico, 00128 Rome, Italy; f.prata@policlinicocampus.it

**Keywords:** NGS analysis, *BRCA1*/2, storage time, target therapy, metastatic castration resistant prostate cancer

## Abstract

Background: Approximately 25.0% of metastatic prostate cancer patients harbour DNA damage repair mutations, including *BRCA1* and *BRCA2*, which are actionable targets for poly(ADP-ribose) polymerase (PARP) inhibitors. Accurate detection of *BRCA1*/2 mutations is critical for guiding targeted therapies, but crucial pre-analytical factors, such as tissue storage duration and DNA fragmentation, drastically affect the reliability of next-generation sequencing (NGS) using real-world diagnostic specimens. Methods: This multicentre study analysed 954 formalin-fixed paraffin-embedded tissue samples from 11 centres, including 559 biopsies and 395 surgical specimens. This study examined the impact of storage duration (<1 year, 1–2 years, and >2 years) and DNA parameters (concentration and fragmentation index) on NGS success rates. Logistic regression and Cox regression analyses were used to assess correlations between these factors and sequencing outcomes. Results: NGS success rates decreased significantly with longer storage, from 87.8% (<1 year) to 69.1% (>2 years). Samples with higher DNA concentrations and fragmentation indexes had higher success rates (*p* < 0.001). Surgical specimens had superior success rates (83.3%) compared with biopsies (72.8%) due to better DNA quality. The DNA degradation rate was more pronounced in older samples, underscoring the negative impact of extended storage. Conclusions: Timely testing of *BRCA1*/2 mutations is critical for optimizing the identification of prostate cancer patients eligible for PARP inhibitors. Surgical specimens provide more reliable results than biopsies and minimizing the storage duration significantly enhances testing outcomes. Standardizing pre-analytical and laboratory procedures across centres is essential to ensure personalized treatments and improve patient outcomes.

## Simple Summary

Timely detection of *BRCA1/2* mutations is essential for identifying metastatic prostate cancer patients who may benefit from PARP inhibitor therapy. However, pre-analytical factors, such as storage time and sample quality, significantly affect sequencing success. In this large multicentre study, we found that prolonged storage time, lower DNA concentration, and biopsy specimens were associated with reduced success rates for BRCA testing. Our findings support the need for early molecular testing and standardized tissue handling protocols to improve patient eligibility for targeted therapies.

## 1. Introduction

Prostate cancer is the second most frequent cancer in men and the fifth leading cause of cancer-related death [1]. Every year, 1,414,259 new diagnoses and 375,304 prostate cancer-specific deaths are reported worldwide [2], with a median overall survival of 22 months in metastatic patients [3]. Up to 25.0% of metastatic prostate adenocarcinomas have somatic alterations in DNA repair genes involved in homologous recombination repair, most notably *BRCA1* and *BRCA2*, along with other genes (e.g., *ATM*, *BARD1*, *BRPIP1*, *MRE11 FANCA*, and *PALB2*) and genes related to genomic instability (e.g., *CDK12*, *CHEK2*, and *MSH2*) [4,5]. Specifically, *BRCA1* and *BRCA2* are broad oncosuppressors located on chromosomes 17q21 and 13q12-q13, containing 22 and 27 exons, respectively [6,7,8].

The clinical relevance of *BRCA1/2* mutations has become evident with the introduction of poly(ADP-ribose) polymerase (PARP) inhibitors. These therapies are well-established in the treatment of ovarian, breast, and pancreatic cancers [9], especially for patients with mutations in DNA damage repair genes, and were approved in 2020 by both the FDA and EMA specifically for the treatment of metastatic castration-resistant prostate cancer (mCRPC) [10,11]. This marked a significant advance in targeted therapy for prostate cancer patients, particularly those with *BRCA1/2* mutations, expanding the clinically available therapeutic options and improving clinical outcomes in these patients [12,13].

Notably, current clinical guidelines lack precise recommendations on the acceptable archival duration for FFPE tissue samples to maintain sequencing success, particularly for *BRCA1/2* analysis. Our study directly addresses this gap by providing real-world multicentre evidence quantifying the impact of storage time on sequencing outcomes.

As a result, next-generation sequencing (NGS) is a key tool for detecting somatic mutations in *BRCA1/2* to identify metastatic prostate cancer patients who could benefit from PARP inhibitor therapies [14,15]. However, the technical sensitivity of NGS in detecting clinically relevant *BRCA1/2* mutations can decrease over time, particularly in formalin-fixed paraffin-embedded (FFPE) tissue samples because of a high DNA fragmentation rate [16] and chemical modifications [17].

Both fragmentation and chemical modifications compromise sequencing fidelity by causing shorter read lengths, impaired polymerase activity, and amplification bias during library preparation, ultimately lowering the reliability of mutation detection.

This study evaluated the sequencing success rate (SSR) of *BRCA1/2* mutations using NGS platforms on FFPE samples from prostate cancer patients stored for various durations and correlated success with the DNA fragmentation rate. We also determined the optimal archival storage timing for *BRCA1*/2 mutation analysis [18,19].

## 2. Materials and Methods

### 2.1. Patients and Tissue Samples

A total of 998 consecutive tissue samples from 11 different institutions with proven experience in *BRCA1/2* molecular analysis were tested (Table S1) to evaluate the detection rate of *BRCA1*/2 mutations by the NGS systems, correlating these data with the duration of archival storage. The FFPE samples had been collected between May 1997 and March 2024. Data collected for each sample included the age of the paraffin block, timing of NGS analysis, type of biopsy specimen, histological diagnosis, DNA fragmentation profile and concentration, DNA extraction kit procedure, and molecular testing platform. The impact of these variables on DNA yield and the SSR of the NGS platform was then evaluated.

After excluding 44 samples (Table S1) because of missing information on the timing of NGS analysis and/or the date of FFPE sample preparation, a total of 954 tissue samples were retrospectively investigated, including 559 (58.6%) biopsy samples (495 from primary sites and 64 from metastatic sites) and 395 (41.4%) surgical specimens (Table S2). The overall mutation rate was 16% (149/954 samples). Based on the duration of archival storage, samples were grouped into three categories [20]:

• Short-term storage (<1 year; 223 biopsies [71.5%] and 89 surgical specimens [28.5%]);
• Middle-term (1–2 years; 86 biopsies [61%] and 55 surgical specimens [39%]);
• Long-term (>2 years; 250 biopsies [49.9%] and 251 surgical specimens [50.1%]).

### 2.2. Molecular Analysis

Molecular and clinical data, as well as additional quality parameters (DNA concentration and fragmentation rate), were retrieved from institutional pathology databases. Panel kits were used to identify single-nucleotide polymorphisms (SNV), insertions–deletions, and/or copy number variations in *BRCA1* and *BRCA2*.

The present study specifically focused on *BRCA1/2* sequencing outcomes, given their direct therapeutic implications for PARP inhibitor eligibility in metastatic prostate cancer patients. Future work could expand the analysis to other DNA repair genes beyond *BRCA1/2* as potential alternative biomarkers.

Sequencing was performed on the MiSeq (Illumina, San Diego, CA, USA) or Ion GeneStudio™ s5 (Thermofisher Scientific, Waltham, MA, USA) platforms [21]. NGS panels allow for the simultaneous analysis of the coding sequence, exon–intron junctions, and the partial 5′ and 3′ untranslated regions of the *BRCA1* and *BRCA2* genes. Molecular testing was performed using various extraction kits and the DNA was analysed using different platforms (Table S3).

### 2.3. Statistical Analysis

Categorical data were reported as frequencies and percentages, and compared using the chi-squared test. To compare SSRs across different time intervals, we conducted pairwise comparisons of proportions. Logistic regression analysis was performed to determine the optimal timing of the *BRCA1/2* mutation analysis, obtaining odds ratios (ORs) and 95% confidence intervals (CIs). Cox regression analysis was conducted to explore the relationship between DNA fragmentation and the SSR for *BRCA1/2* mutation detection, obtaining hazard ratios (HRs) and 95% CIs. The chi-squared test was used to examine the association between SSR and the type of tissue sample and biopsy site. The significance threshold was set at a two-sided *p*-value < 0.05. The Cox proportional hazards regression model was employed to assess the associations of DNA concentration, DNA fragmentation, and sample type with SSR. The proportional hazards assumption was tested using Schoenfeld residuals, reported as HRs with 95% CIs. The log-rank test was used to assess differences in the SSR distributions between biopsies and surgical specimens. All statistical analyses were performed using STATA (StataCorp, 2021 Stata Statistical Software: Release 17, College Station, TX, USA: StataCorp LLC) and SPSS (IBM SPSS Statistics for Windows, Version 28.0: IBM Corp., Armonk, NY, USA).

## 3. Results

### 3.1. Success Rate and Storage Time

**Figure 1:**
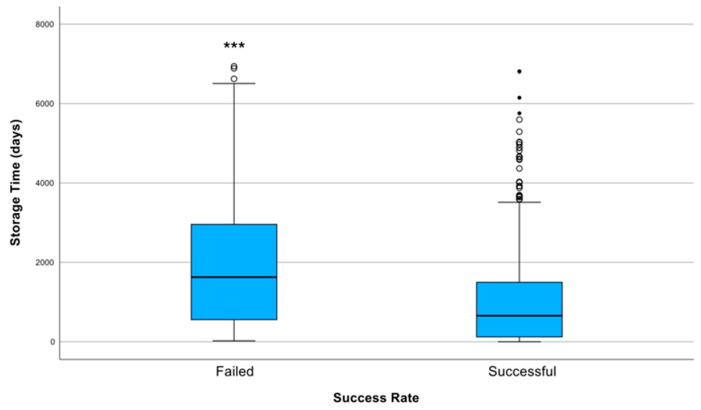
Distribution of storage duration (in days) according to sequencing success rate (SSR). Successfully sequenced samples (SSR = 1) had a significantly shorter median storage time (657 days; IQR: 122.5–1498) compared with failed samples (SSR = 0), which had a median storage duration of 1626.5 days (IQR: 557–2956). The difference in storage times between groups was statistically significant (*** indicates *p* < 0.001 (Mann–Whitney U test)). The failed group also exhibited greater variability and more extreme outliers, indicating that longer storage durations were associated with sequencing failure.

The overall SSR of DNA sequencing across all samples was 77.1% (736/954). Specifically, we found that failed samples had a longer storage duration than successful samples; the median storage time of failed samples was 1626.5 days (4 years and 5 months) (interquartile range [IQR], 557–2956 days), whereas for successfully analysed samples, it was 657 days (IQR, 122.5–1498 days). These results were significantly different according to the Mann–Whitney test (*p* < 0.001; Figure 1).

**Figure 2:**
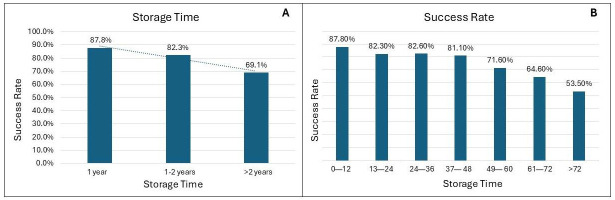
(**A**) DNA sequencing success rate (SSR) according to categorical storage duration. SSR was highest in samples stored for <1 year (87.8%; 274/312) and 1–2 years (82.3%; 116/141), compared with >2 years (69.1%; 346/501). Long-term storage (>2 years) was significantly associated with a reduced SSR (OR = 0.36; 95% CI: 0.26–0.50; *p* < 0.001), whereas short-term storage (<1 year) was significantly associated with a higher SSR (OR = 2.80; 95% CI: 1.92–4.11; *p* < 0.001). Pairwise proportion tests confirmed significant differences between long-term storage and both short-term (*p* < 0.001) and middle-term (*p* = 0.002) storage, while no significant difference emerged between short- and middle-term storage (*p* = 0.114). (**B**) SSR stratified by storage duration in 12-month intervals, showing a progressive decline over time. The SSR dropped from 87.8% (0–12 months) to 53.5% (>72 months).

Storage duration had a notable effect on SSR. Long-term storage (>2 years) was associated with a significant reduction in the SSR (OR, 0.36; 95% CI, 0.26–0.50; *p* < 0.001), indicating an inverse correlation between storage time and SSR. Conversely, short-term storage (<1 year) was significantly associated with a higher SSR (OR 2.8, 95% CI 1.92–4.11, *p* < 0.001). As depicted in Figure 2A, SSR was 87.8% (274/312) for samples stored for <1 year, 82.3% (116/141) for those stored between one and two years, and it dropped to 69.1% (346/501) for samples stored >2 years. Pairwise proportion tests revealed significant differences in success rates across storage time intervals. The SSR after 2 years was significantly lower than the SSR for short-term (69.1% vs. 87.8%, *p* < 0.001) and middle-term storage samples (69.1% vs. 82.3%, *p* = 0.002). The difference between samples stored short-term and middle-term (87.8% vs. 82.3%) was not significant (*p* = 0.114). These findings highlight a notable decline in the SSR over time, particularly beyond 2 years of storage.

This declining trend is further detailed in Figure 2B, which breaks down the SSR over 7-year intervals, showing a consistent reduction in the SSR with increasing length of storage. For example, the SSR was highest for samples stored between 0 and 12 months, and the SSR dropped to 50% for samples stored for more than 72 months.

**Figure 3:**
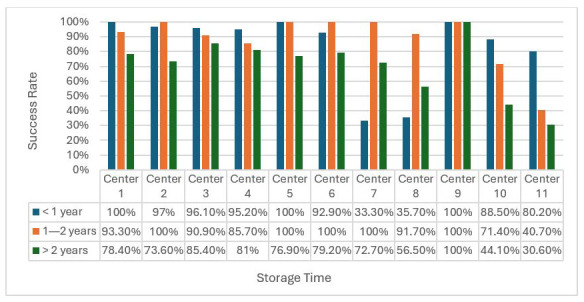
Success rates across different centres stratified by follow-up time (<1 year, 1–2 years, and >2 years). Bars represent the percentage of samples with successful sequencing (SSR = 1) at each time point. While success rates were generally high at early time points, marked variability was observed at >2 years, indicating differences in long-term sample quality among centres. No statistical comparisons were performed between centres. Bar heights represent the proportion of successfully sequenced samples. Data labels indicate exact success percentages.

Examining each participating institution (Figure 3), we observed a remarkable decrease in the SSR of molecular analysis for samples stored >2 years. Notably, few centres presented a distinct pattern, with particularly low SSRs for short-term storage and higher SSRs for middle-term storage. This difference could be due to sample size or specific pre-analytical variables.

### 3.2. Success Rate and DNA Concentration and Fragmentation

We found that DNA concentration and fragmentation were also related to the SSR. Higher DNA concentrations were positively correlated with the SSR (OR, 1.13; 95% CI, 1.09–1.16; *p* < 0.001), suggesting that an adequate DNA concentration enhances the reliability of DNA sequencing. In particular, there was a significant difference between failed and successful samples (*p* < 0.001), confirming higher concentrations in successfully tested samples compared with failed samples (Figure S1). We also found a significant correlation between DNA fragmentation and the SSR (OR, 4.24; 95% CI, 3.05–5.90; *p* < 0.001), as successful samples had a higher fragmentation index than failed samples. It is important to clarify that, the DNA fragmentation index is inversely correlated with DNA degradation: a higher fragmentation index indicates better DNA integrity, and thus a higher likelihood of sequencing success.

### 3.3. Success Rate and Type of Sample

We observed a significant difference in storage duration between biopsies and surgical specimens. Biopsies had a shorter median storage time (596 days; IQR, 118–1402) compared with surgical specimens, which had a median storage time of 1287 days (IQR, 464–2321; *p* < 0.001; Figure S2).

Moreover, regarding success rate (SSR), surgical specimens demonstrated a significantly higher SRR compared with biopsies (*p* < 0.001; Table 1). Within the biopsy group, no significant differences in SRR were identified based on the biopsy site, even when considering bone biopsies (*p* = 0.25).

The analysis of median DNA concentrations and DNA fragmentation values revealed notable differences between biopsies and surgical specimens. The DNA concentration was significantly higher in surgical specimens (13.05 ng/µL; IQR, 3.25–39.35) than in biopsies (2.2 ng/µL; IQR, 0.46–7) due to the larger tissue volume and greater sample yield from surgical specimens. Similarly, DNA fragmentation values demonstrated a consistent trend, with a higher median fragmentation rate in surgical specimens (1.2; IQR, 0.29–2.37) compared with biopsies (0.41; IQR, 0.19–1.7).

Furthermore, the SSRs for biopsies and surgical specimens were analysed over a 3-year period to assess trends over time (Table S4). For short-term storage, the biopsy SSR was 84.3%, whereas the surgical specimen SSR was significantly higher (*p* = 0.003) at 96.6%. For middle-term storage, the biopsy SSR significantly decreased to 73.3% (*p* < 0.001), but the surgical specimen SSR remained high at 96.4%. The difference between the two groups remained significant with long-term storage (*p* = 0.001); the biopsy SSR was 62.4% and the surgical specimen SSR 75.7%.

### 3.4. Multivariate Analysis

**Figure 4:**
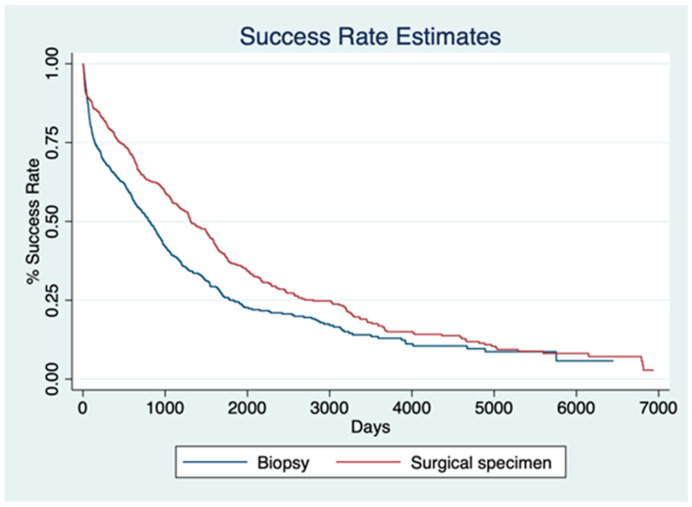
Kaplan–Meier curves illustrating success rate (SSR) estimates over time for biopsy (blue) and surgical specimen (red) groups. Patients with biopsy samples had a significantly lower hazard for SSR compared with those with surgical specimens (HR = 0.7362; 95% CI: 0.5810–0.9328; *p* = 0.011). The log-rank test confirmed a significant difference in SSR distributions between the two cohorts (*p* < 0.001).

Cox regression analysis demonstrated that both high DNA concentration and high DNA fragmentation index were significantly associated with a higher SSR. Specifically, each unit increase in DNA concentration was associated with a 0.35% increase in SSR (HR, 1.0035; 95% CI, 1.0024–1.0046; *p* < 0.001), whereas a higher DNA fragmentation index was associated with a 26.6% increase in SSR (HR, 1.2661; 95% CI, 1.1479–1.3965; *p* < 0.001). Patients with biopsy samples had a significantly lower hazard for SSR compared with surgical specimens (HR, 0.7362; 95% CI, 0.5810–0.9328; *p* = 0.011). Finally, the log-rank test demonstrated a significant difference in SSR distributions between biopsy and surgical specimen cohorts (*p* < 0.001) (Figure 4).

## 4. Discussion

Our study revealed that prostate cancer samples stored for a short time had a markedly greater success rate in detecting *BRCA1/2* mutation compared with samples stored long-term. This difference is further supported by the logistic regression analysis, indicating that short-term storage significantly increases the likelihood of successful sequencing by 2.8-fold (*p* < 0.001), whereas long-term storage is associated with a substantial reduction in the SSR (OR, 0.36; *p* < 0.001). In addition, the detailed breakdown of SSR over 7-year intervals reinforced the inverse relationship between storage duration and SSR that progressively declined over time.

Despite the advent of the NGS era, detecting clinically relevant *BRCA1/2* mutations remains a significant challenge, primarily due to DNA fragmentation derived from pre-analytical processing and storage procedures. The DNA degradation rate can affect the availability of biological material for molecular testing, leading to significantly increased failure rates, potentially denying access to treatments for prostate cancer patients. The PROfound study, which evaluated the efficacy of comprehensive gene profiling (CGP) in detecting *BRCA1/2* somatic mutations, highlighted technical challenges to successfully implementing CGP in diagnostic routine practice for prostate cancer patients. CGP failed to provide reliable results in 42.0% of cases, resulting in SSRs of 63.9% for newly obtained tissue samples and 56.9% for archival samples [18]. Moreover, the reported discordance rate between CGP and in-house molecular testing methods raises concerns about the consistency and reliability of these testing strategies in clinical practice [22]. Despite CGP offering a comprehensive overview of molecular fingerprinting of tumour patients, the variability in terms of SSR impacts the consistency of molecular results across different testing platforms. Notably, *BRCA1/2* testing approaches for real-world samples can be further affected by challenging factors, including sample quality, the expertise of the personnel, and the lack of harmonized procedures. As such, there is a need to better understand the variables that can affect the SSR of *BRCA1/2* testing. These findings suggest that prolonged archival storage negatively impacts DNA fragmentation, excluding patients who could benefit from targeted therapies [23].

Analysis of individual centres revealed notable heterogeneity in patterns of success, particularly for short-term and middle-term storage samples. Although the vast majority of institutions demonstrated a similarly decreasing SSR for long-term storage, some cases exhibited an anomalously lower SSR for short-term storage and higher SSR for middle-term storage. These discrepancies may stem from centre-specific pre-analytical variables, such as sample collection, preservation methods, or storage conditions. Similar results were obtained by Tommasi et al. [20], who reported discordance in CGP results across different laboratories, underscoring the need for standardized protocols to minimize variability and ensure reliable results across testing platforms.

Furthermore, our study revealed a positive correlation between DNA concentration and SSR (OR, 1.13; *p* < 0.001), indicating that even marginal increases in DNA concentration significantly enhance the likelihood of successful sequencing analysis. This substantial disparity reflects the consistently higher DNA concentration observed in successfully analysed samples compared with failed cases. The implications of our findings extend to practical considerations in sample preparation. Ensuring that the DNA concentration exceeds a minimum threshold is crucial for reliability in sequencing [24].

The DNA fragmentation index also emerged as a significant predictor of successful sequencing strategies [25]. Logistic regression analysis revealed a significant correlation between higher fragmentation index and increased SSR (OR, 4.24; *p* < 0.001). Logistic regression analysis revealed a strong positive correlation between higher fragmentation index values, reflecting better DNA integrity, and increased SR (OR = 4.24; *p* < 0.001). Integrating storage time, DNA concentration, and fragmentation index clearly presents how pre-analytical procedures play a pivotal role in guiding the success of analytical strategies. Longer archival storage times are associated with decreased SSRs due to progressive DNA degradation. These findings align with those from Capoluongo et al. [26], who demonstrated that accurate quantification techniques, such as fluorometric assays, are crucial for reliable sequencing outcomes. In our study, marginal increases in DNA concentration significantly boosted the SSR, highlighting the importance of achieving and maintaining an optimal concentration during library preparation. Similarly, PROREPAIR-B pointed out the importance of high-quality DNA for reliably detecting homologous recombination repair mutations in castration-resistant prostate cancer, further validating the need for robust pre-analytical handling protocols [27]. Leith et al. [28] highlighted the critical role of high-quality DNA and robust protocols in optimizing molecular testing for metastatic prostate cancer. They emphasized the importance of proper tissue handling, advanced DNA quantification techniques, and standardized storage to mitigate degradation and variability [29]. This study stresses the need for validating protocols across clinical settings to reduce inconsistencies and improve reliability. These measures are essential for identifying prostate cancer patients eligible for PARP inhibitors [30].

Our study highlights a significant correlation between sample type and the SSR for sequencing *BRCA1/2*. Surgical specimens exhibited a pattern of significantly greater success compared to biopsies, emphasizing their greater reliability for molecular testing, whereas both types of sampling exhibit a decline in accuracy as storage time increases. Notably, differences between biopsies and surgical specimens strictly depend on the quality of the extracted DNA. In this study, surgical specimens exhibited significantly higher DNA fragmentation indexes compared with biopsies (13.05 vs. 2.2), which reflects better DNA integrity and overall quality.

The substantial reduction in SSR using biopsy specimens highlights the greater reliability of surgical specimens for the detection of *BRCA1/2* mutations. To the best of our knowledge, no similar data have been reported for prostate cancer specimens. Variations in formalin fixation time across different centres and specimens may also contribute to these differences.

Taken together, these findings have important implications for clinical practice. Although the detrimental impact of prolonged tissue storage on nucleic acid quality is widely acknowledged, our study provides a real-world, multicentre validation specifically within the context of *BRCA1/2* testing in metastatic prostate cancer on a large cohort of samples. Our findings, beyond confirming an intuitive biological principle, emphasize the urgent need for defining acceptable archival time limits and standardizing pre-analytical workflows across institutions to optimize molecular diagnostics in real-world settings. Moreover, sequencing failure due to DNA degradation or prolonged storage may preclude eligible patients from accessing life-prolonging PARP inhibitor therapy, underscoring the real-world clinical implications of pre-analytical quality.

### Limitations

Our study has several limitations. A significant limitation is the retrospective collection of data, which restricts control over variables such as pre-analytical handling, storage conditions, and DNA extraction protocols. Due to the retrospective and multicentre nature of this study, detailed pre-analytical information such as fixation time, fixation temperature, storage environment, and FFPE processing protocols could not be systematically retrieved across all centres.

In addition, the variability in SSR across different centres suggests that pre-analytical variables significantly impact the results; standardization across different centres can help to mitigate this issue. Moreover, although this study highlights the importance of DNA concentration and fragmentation, it may not fully account for other aspects of DNA quality that can also affect sequencing success.

Despite its limitations, this study offers a large real-world perspective on the importance of addressing practical challenges encountered in everyday clinical settings in a multicentre study, making our findings directly applicable for improving laboratory practices.

## 5. Conclusions

Our findings highlight that minimizing storage duration, and improving and prioritizing higher-quality DNA samples can significantly enhance the success rate of DNA sequencing, reinforcing observations from prior studies on other cancer types, such as breast and ovarian cancers. Moreover, our findings reinforce the importance of applying established pre-analytical best practices—already validated in other tumour types—to *BRCA1/2* testing in prostate cancer. Moreover, we underline the role of the pathologist in selecting the sample for molecular analysis to favour surgical specimens rather than biopsy specimens when both are available. The novel approach to systematic *BRCA1/2* mutation testing represents a paradigm shift in prostate cancer management. Confined thus far to metastatic cases, our findings support the concept of early *BRCA1/2* mutation screening in high-risk and very-high-risk patients. This new strategy not only improves the ability to detect *BRCA1/2* mutations, but lays the foundation for highly personalized and precisely tailored treatment, leading to better patient outcomes.

## Figures and Tables

**Table 1:** Comparison of sequencing analysis outcomes for biopsies and surgical specimens.

Outcome of Analysis	Biopsies	Surgical Specimens
Failed	152/559 (27.2%)	66/395 (16.7%)
Successful	407/559 (72.8%)	329/395 (83.3%)

## Data Availability

The data presented in this study are available on request from the corresponding author.

## Supplementary Materials

The following supporting information can be downloaded at: https://www.mdpi.com/article/10.3390/cancers17101705/s1, Table S1: Distribution of cases across various medical centers. Cases were excluded due to missing information on the timing of next-generation sequencing analysis and/or the date of sample preparation. Table S2: Breakdown of tissue samples analyzed in the study. Table S3: The study utilized various DNA extraction kits and sequencing platforms to analyze BRCA1 and BRCA2 mutations in FFPE tumor tissues. DNA sequencing was performed using advanced kits and platforms. Table S4: Comparison of success rates (SRs) between biopsies and surgical specimens across different storage durations. Figure S1: Comparison between groups showing that failed samples (N = 210) had a lower mean rank of concentration (188.28 ng/µL), whereas successful samples (N = 495) had a higher mean rank of concentration (422.88 ng/µL). The x-axis represents frequency, with failed samples on the left and successful samples on the right, whereas the y-axis represents concentration. Figure S2: Comparison of the storage time (in days) between two types of samples: biopsy and surgical specimen. Biopsy samples had a lower median storage time and reduced variability than surgical specimens. Surgical specimens had more extreme outliers.

## Abbreviations

The following abbreviations are used in this manuscript:

NGS	Next-Generation Sequencing
PARP	Poly(ADP-ribose) Polymerase
BRCA	Breast Cancer Gene
FFPE	Formalin-Fixed Paraffin-Embedded
