# Supplementary material for: Storage Time and DNA Quality Determine BRCA1/2 Sequencing Success in Prostate Cancer: A Multicentre Analysis with Therapeutic Implications
Source: Cancers (Basel). 2025 May 20;17(10):1705. doi: 10.3390/cancers17101705 (PMC12110138; doi:10.3390/cancers17101705)
Supplement: Supplementary file 1 [file cancers-17-01705-s001.zip › cancers-3604425-supplementary.pdf]

Supplementary materials

Centers	No. of cases	No. excluded cases	No. analyzed cases
Roma-IFO	84	6	78
Bologna	101	1	100
Roma-UCBM	180	0	180
Milano-IEO	54	2	52
Roma-Sant'Andrea	27	16	11
Napoli-Federico II	86	1	85
Monza	28	13	15
Bari	57	1	56
Salerno	51	2	49
Firenze	261	0	261
Treviso	69	2	67
TOTAL	998	44	954

Supplementary Table S1: Distribution of cases across various medical centers. Cases were excluded due to missing information on the timing of next-generation sequencing analysis and/or the date of sample preparation.

Category	Tissue type	No. of samples	Percentage of total samples
**Biopsy samples**	Prostate	495	51.90%
	Lymph nodes	21	2.20%
	Bone	14	1.50%
	Liver	9	0.90%
	Bladder	7	0.70%
	Lung	8	0.80%
	Colon	2	0.20%
	Abdominal wall	2	0.20%
	Meninges	1	0.10%
**Subtotal**		**559**	**58.60%**
**Surgical specimens**	Total surgical samples	**395**	**41.40%**
**Total**		**954**	**100%**

Success rate

Supplementary Table S2: Breakdown of tissue samples analyzed in the study.

EXTRACTION KIT
QIAGEN EZ1 & EZ2 DNA Tissue Kit (Cat no. 953034)
MagCore genomic DNA FFPE (Cat no. 405/MGF-01)
MagMAX™ FFPE DNA/RNA Ultra Kit (Cat no. A31881)
MAXWELL CSC DNA FFPE (Cat no. AS1350)
QIAGEN Mini Amp Kit (Cat no. 51304)
QIAamp DNA FFPE Tissue Kit (Cat no. 56404)
QuickExtract FFPE DNA Extraction Kit (Cat no. QEF81050)
**ANALYSIS KIT**
Myriapod (NGS platform: Illumina) (Cat no. NG035)
AmoyDX Focus (NGS platform: Illumina) (Cat no. 8.06.0047)
Oncomine Tumor-Specific Panel, Thermo Fisher Scientific - Custom Panel (PMID: 37240284) (NGS platform: Thermo Fisher Scientific)

Supplementary Table S3: The study utilized various DNA extraction kits and sequencing platforms to analyze BRCA1 and BRCA2 mutations in FFPE tumor tissues. DNA sequencing was performed using advanced kits and platforms.

Storage time	Biopsy SR	Surgical specimen SR	p-value
< 1 year	188/223 (84.3%)	86/89 (96.6%)	0.003
1-2 years	63/86 (73.3%)	53/55 (96.4%)	<0.001
>2 years	156/250 (62.4%)	190/251 (75.7%)	0.001

Supplementary Table S4: Comparison of success rates (SRs) between biopsies and surgical specimens across different storage durations.

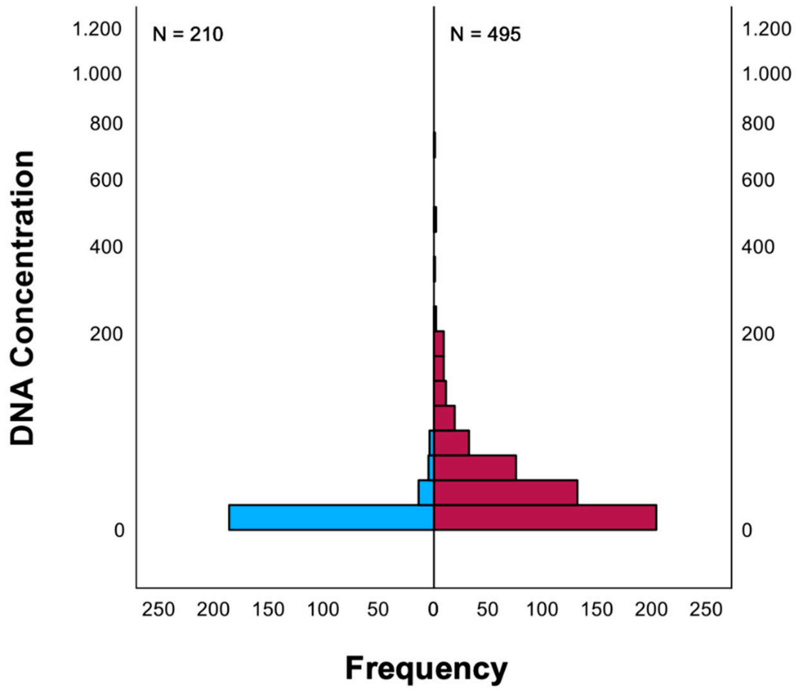

Supplementary Figure S1: Comparison between groups showing that failed samples (N=210) had a lower mean rank of concentration (188.28 ng/µL), whereas successful samples (N=495) had a higher mean rank of concentration (422.88 ng/µL). The x-axis represents frequency, with failed samples on the left and successful samples on the right, whereas the y-axis represents concentration.

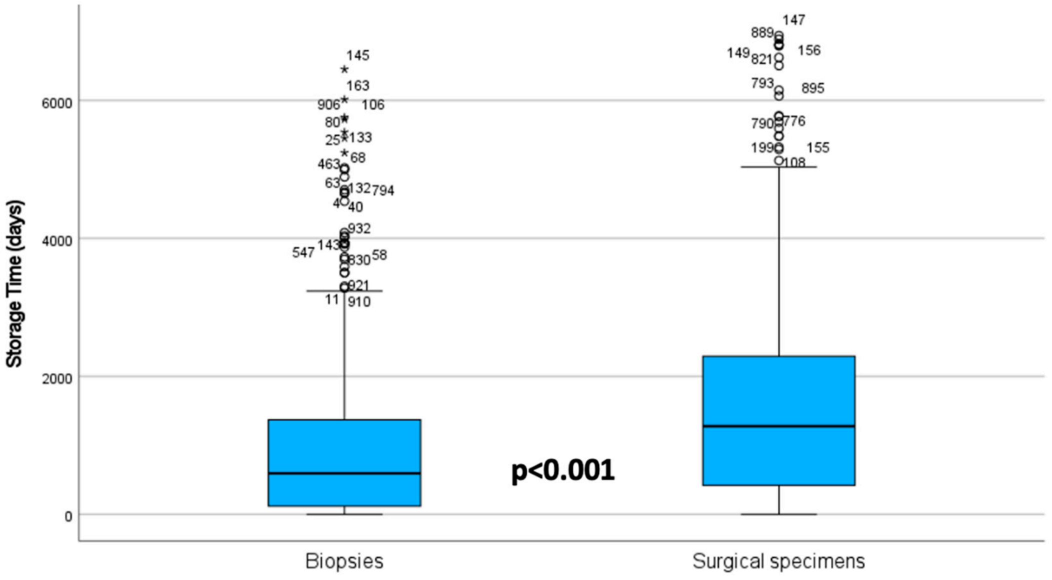

Supplementary Figure S2: Comparison of the storage time (in days) between two types of samples: biopsy and surgical specimen. Biopsy samples had a lower median storage time and reduced variability than surgical specimens. Surgical specimens had more extreme outliers.
